# Tap Water Consumption and Perceptions in United States Latinx Adults

**DOI:** 10.3390/nu13092999

**Published:** 2021-08-28

**Authors:** Abigail T. Colburn, Stavros A. Kavouras

**Affiliations:** Hydration Science Lab., College of Health Solutions, Arizona State University, Phoenix, AZ 85004, USA; atcolbur@asu.edu

**Keywords:** hydration, total water intake, plain water intake, tap water, bottled water, Latino adults, Hispanic adults

## Abstract

Insufficient water intake is associated with adverse health outcomes, including chronic disease prevalence and mortality. Adherence to Institute of Medicine total water intake (TWI) recommendations has been low in recent decades, and TWI has been consistently lower in Latinx adults compared with non-Hispanic (NH) white adults. While overall plain water intake is similar between Latinx and NH white adults, Latinx adults consistently consume significantly more bottled water and less tap water. The purpose of this review is to identify factors that may contribute to low water intake and low tap water intake, particularly in Latinx adults. The decision to drink water is complex and is influenced by a myriad of factors including context, environment, eating behaviors, geography, and beverage attributes. Plain water preferences appear to be related, in part, to perceptions of tap water safety as Latinx adults are significantly more likely to perceive their tap water as unsafe compared to NH white adults. Although recent investigations have not consistently or comprehensively evaluated the same factors, we have compiled their findings to describe the complex, interrelated determinants of tap water safety perceptions in Latinx adults. The present review proposes that perceptions are influenced by water insecurity, demographics, prior experiences, organoleptic (sensory) perceptions and availability and sources of information. Existing interventions designed to improve TWI primarily focus on improving access to water and/or educating individuals on the importance of hydration. However, this may not be sufficient in Latinx populations where water is not trusted. Future work should comprehensively assess these factors in Latinx samples and include validated plain water intake, TWI, and hydration status measures. A greater understanding of these relationships could inform interventions to improve TWI and hydration status in Latinx adults.

## 1. Hydration and Water Intake

### 1.1. Hydration and Health

The Institute of Medicine (IOM) recommends adult men and women in the United States (US) consume 3.7 L and 2.7 L per day, respectively, to maintain euhydration [1]. These recommendations for total water intake (TWI) can be met through plain water intake (PWI; water consumed via tap water and bottled water) as well as through water consumed via beverages and foods [1]. Water needs can vary between and within individuals due to factors such as physical activity level, environmental conditions (i.e., ambient temperature, humidity), diet (i.e., solute load, macronutrient composition), and body composition [1]. Insufficient water intake can lead to underhydration (i.e., stimulation of water conservation mechanisms without changes in total body water) and dehydration (i.e., stimulation of water conservation mechanisms with deficits in total body water) [2]. 

Underhydration, dehydration, and low water intake are consistently associated with adverse health outcomes. A recent cross-sectional analysis of the nationally representative National Health and Nutrition Examination Survey (NHANES) reported a significantly greater prevalence of obesity, high waist circumference, insulin resistance, low high-density lipoprotein, and metabolic syndrome in underhydrated compared to hydrated adults (51–70 y). The prevalence of those suffering no chronic health conditions in this sample was significantly lower. In contrast, chronic disease mortality was estimated to be 4.2 times greater in underhydrated adults than in euhydrated adults [3]. The IOM has similarly linked dehydration to numerous health outcomes, including cardiovascular dysfunction, urinary tract infections, several chronic diseases, and death [1]. Moreover, low water intake has been associated with chronic kidney disease and diabetes [4,5,6], while increased water intake has been associated with positive health outcomes, including augmented cognitive performance in children [7], less frequent urinary tract infections [8], and enhanced glucose regulation [9]. 

Population-level TWI and PWI in adults ≥ 20 y have been estimated from numerous recent NHANES cohorts: 2005–2010 cycles (*n* = 15,702) [10], 2009–2012 cycles (*n* = 8258) [11], 2011–2014 cycles (*n* = 9666) [12], and 2011–2016 NHANES cycles (*n* = 15,263) [13]. All water intake values were estimated from 24 h dietary recall interviews and data are reported as mean ± standard error [10,11,12]. The United States Department of Agriculture’s automated multiple-pass method was utilized to conduct these 24 h dietary recalls, which is a validated method for energy and nutrient intake [14]. Although this method has not been validated for TWI or PWI, it includes many mechanisms to help with accuracy of reporting (e.g., the provision of visual cues for estimating food and beverage amounts and reminders for interviewers about missing data [15]). Average TWI and adherence to IOM adequate intake recommendations from 2005 to 2010 [10] and 2011 to 2016 [13] are presented in Table 1. Average TWI was similar for adults > 70 y across both time periods but appeared to decrease for younger adults in more recent years. Adherence to IOM recommendations was low across all age groups, with the lowest prevalence in those > 70 y. Moreover, adherence was more prevalent in women than men across all age groups. 

### 1.2. Hydration Status and TWI in Latinx Adults

TWI has been consistently lower in Latinx adults compared to non-Hispanic (NH) white adults (Table 2) [10,11,12]. Across 2009–2012 specifically, average TWI was significantly lower among Hispanic than NH white adults by 341 mL (95% CI = −472, −209 mL) [11]. Accordingly, Hispanic adults were 1.42 times (95% CI = 1.21, 1.67) more likely to be inadequately hydrated (spot urine osmolality > 800 mmol·kg^−1^) compared to NH white adults [11]. Interestingly, the odds of inadequate hydration were slightly lower for Hispanic adults who consumed any tap water (odds ratio [OR] = 1.37; 95% CI = 1.18, 1.59) [11]. For the entire sample, 29.5% of individuals were inadequately hydrated, and the risk of inadequate hydration was lower for adults consuming any tap water (OR = 0.83; 95% CI = 0.70, 0.98) [11]. 

While there is no gold standard marker of hydration status, it has been recommended to incorporate multiple markers to determine a more accurate assessment [16]. Adults 51–70 y from the same NHANES cohorts (2009–2012) were classified as underhydrated based on serum sodium > 145 mmol·L^−1^, spot urine volume < 50 mL, and/or spot urine osmolality ≥ 500 mmol·kg^−1^ [3]. When utilizing these markers, an estimated 69.4% of the sample was underhydrated [3]. Moreover, a urine osmolality cut-off of 500 mmol·kg^−1^ may be more appropriate than a cut-off of 800 mmol·kg^−1^, as antidiuretic mechanisms have been observed to be activated via elevated plasma osmolality when urine osmolality is 500–800 mmol·kg^−1^ [17]. Therefore, discrepancies in risk for underhydration and associated deleterious health outcomes may be greater than currently reported. 

Overall PWI has mostly been similar between NH white and Latinx adults (Table 2). While PWI was significantly greater in Hispanic adults than NH white adults from 2011–2014 [12], 120 mL (~4 oz) of water is not a clinically meaningful difference. Interestingly, sources of PWI have consistently been different between Latinx and NH white adults. Among all adults from 2005–2010, 56.0% of PWI came from tap water [10]. NH white adults consumed the most tap water and least bottled water compared to Mexican American and Other Hispanic adults who consumed the least tap water and most bottled water [10]. Similarly, from 2009–2012, Hispanic adults consumed a significant 1.38 fewer servings of tap water (−326 mL; 95% CI = −1.86, −0.54 servings) compared with NH white adults [11]. Conversely, Hispanic adults consumed a significant 1.29 more servings of bottled water (306 mL; 95% CI = 0.83, 1.75) compared with NH white adults [11]. Across 2011–2014, tap water comprised 62.2% of PWI for all adults [12]. Compared to NH white adults, Hispanic adults were significantly less likely to consume tap water (OR = 0.55, 95% CI = 0.45, 0.66) and significantly more likely to consume bottled water (OR = 2.37, 95% CI = 1.79, 2.69) [12]. Furthermore, compared to NH white adults, tap water intake was significantly lower (*B* = −180 ± 64 mL; *p* < 0.05) and bottled water intake was significantly greater (*B* = 243 ± 42 mL; *p* < 0.01) in Hispanic adults [12]. While overall PWI is similar, Latinx adults are particularly averse to tap water. 

The purpose of this review is to identify factors that may contribute to low water intake and low tap water intake, particularly in Latinx adults. The PubMed database was utilized to search for potential research articles related to the current topics: (1) voluntary low total water intake and (2) tap water safety perceptions in US Latinx adults. Articles were included for voluntary low total water intake if they evaluate physiological, social, and/or behavioral cues related to water and/or beverage consumption. Articles that identify characteristics of individuals who consume low volumes of water were also included. Exercise- and physical activity-related articles were excluded. Quantitative articles were included for tap water safety perceptions in US Latinx adults if their primary outcome is drinking water perceptions. Articles were excluded if the sample did not include adults ≥ 18 y, if the study was conducted outside of the United States, or if the sample did not include Latinx individuals. Additional articles were included to provide context to these findings if the sample includes US Latinx individuals and/or if the article focused on a specific aspect of water perceptions (e.g., organoleptic perceptions).

## 2. Voluntary Low Total Water Intake

Water losses occur continuously throughout the day, while fluid intake is episodic and deliberate. Voluntary dehydration can occur when individuals delay compensating for water losses despite access to water [18]. While voluntary dehydration has been described in relation to stressors that accentuate water losses (e.g., physical activity and environmental heat stress), it is evident from NHANES data that a similar phenomenon occurs in the absence of stressors or water deficits that allows for underhydration. 

Thirst has been believed to be a sufficient stimulus to maintain water balance via fluid intake in daily life [19]. However, vasopressin secretion is more sensitive to changes in plasma osmolality than thirst activation. Accordingly, vasopressin will induce water conservation mechanisms (e.g., decreased urine output and increased urine concentration) to regulate plasma osmolality before thirst is needed to prompt water consumption in underhydrated individuals [20]. Furthermore, thirst may not lead to adequate fluid replacement. Swallowing while consuming fluids can activate oropharyngeal receptors and subsequently terminate drinking prematurely via inhibition of vasopressin secretion and thirst despite elevated plasma osmolality [21]. 

### Plain Water Intake

The decision to drink is influenced by context and environment. Regarding beverage consumption in general, adults in rural south-west Virginia believed their behaviors were impacted by time of day, food consumption (e.g., beverage choice depends on food choice), location (e.g., greater likelihood of choosing a sugar-sweetened beverage when going out to eat), time of week (e.g., drinking behaviors are different on weekends compared to weekdays), availability or convenience of a beverage, and the behaviors of other members living in their household [22]. Similarly, the ability of university students to acutely (daily across the upcoming week) choose plain water over sugar-sweetened beverages appears to be predicted by behavioral confidence (i.e., the ability to choose plain water while eating out, while watching TV or sports, and without missing caffeine or carbonation) and changes in the physical environment (i.e., the ability to remove sugar-sweetened beverages from their physical environment, choose water when around someone consuming sugar-sweetened beverages, and choose to purchase water instead of sugar-sweetened beverages) [23]. Regarding water consumption specifically, more than two thirds of plain water is consumed at home [24]. Experiments with vagotomized rats suggested that eating may activate physiological signals for thirst and drinking [25]. However, previous NHANES data have shown that 73.0% of water was consumed outside of meals, with the least amount consumed during breakfast (6.0%) [24]. Moreover, more than half of plain water was consumed independently (with no other food or beverages) [24]. Interestingly, worksite beverage environment (i.e., quantity of water coolers, water fountains, vending machines, and regular soda slots) has not been observed to impact overweight or obese employees’ water consumption [26]. 

Additional factors likely influence the decision to drink plain water, such as age, geographic location, and health-related behaviors and attitudes. Specifically, low plain water intake is more likely in adults ≥ 55 y, adults living in the US Northeast, and adults who are not trying to change their weight (compared to adults trying to lose weight) [27]. Low plain water intake has also been associated with unhealthful behaviors such as moderate physical activity <150 min·wk^−1^ [24,27] and fruit or vegetable consumption ≤1 c·d^−1^ [27]. Similarly, consumption of any plain water was observed in US adults with healthful eating patterns (i.e., greater consumption of fruits, vegetables, and low- and medium-fat dairy products) whereas consumption of no water was observed in adults with unhealthful eating patterns (i.e., high consumption of desserts, high-fat meats, non-caloric and caloric beverages, high-fat dairy, salty snacks, and fast food) [28]. 

Finally, the decision to drink plain water is related to beverage attributes, preferences, and habits. Perceived health outcomes were identified as both positive (e.g., helps body, flushes kidneys, keeps you hydrated, refreshing, and helps metabolism) and negative (e.g., health complications associated with drinking too much water and perceptions that cancer is related to water intake) attributes of water [22]. Concern was expressed regarding chemicals or contamination of water sources, particularly due to fear from health department letters [22]. Taste and cost can similarly be both a positive and negative beverage attribute for water [22]. Taste as a negative attribute may be related to municipal water treatment (e.g., city water was described as bleach water) while cost as a negative attribute was commonly described in reference to bottled water [22]. Taste may be the most influential factor as some have expressed that they would only choose a cheaper beverage if taste was not compromised. Others described their preference for sugar-sweetened beverages as an addiction, which made taste more important than a health risk assessment [29]. Additional preferences may serve as barriers to increasing water intake including water temperature and availability of other options, such as sugar-sweetened beverages [22]. 

## 3. Tap Water Safety Perceptions in US Latinx Adults

Voluntary low TWI is exacerbated in Latinx adults and appears to be driven by tap water avoidance. There are many factors that could influence PWI source preferences (e.g., tap vs. bottled). Adults in rural south-west Virginia identified the availability of their preferred source of water as a barrier to increasing water intake [22]. Contrarily, water intake was supported by the availability and convenience of water (e.g., “Because it’s handy. I always have at least one case of bottled water in the house.”) [22]. During focus group interviews, participants living in an under-resourced rural area in New Mexico reported that convenience was an important influence on PWI source choices, independent of access to safe tap water [30]. Specifically, bottled water was described as easily accessible and transportable, and it can be put in the freezer to accommodate palatability preferences [30]. In a sample of parents of children in an urban/suburban pediatric emergency department, the odds of primarily relying on bottled water were significantly greater with beliefs that bottled water is more convenient (OR = 1.72, 95% CI = 1.16–2.54) than tap water [31]. Furthermore, Latino parents (16.0%) were more likely to endorse a higher level of agreement with the statement “Bottled water is more convenient than tap water” compared to non-Latino white parents (10.6%) (*p* < 0.001) [31]. 

While bottled water is a costly alternative to tap water, income level has not influenced bottled water preference. Bottled water sales have increased in recent years by 34.40% from 2006–2015 [32] and an additional 7.00% from 2016–2017 [33]. Consequently, bottled water has become the most consumed packaged beverage in the US, with bottled water revenues reaching $18.5 billion in 2017 [33]. Based on the 2015 national average price for bottled water ($0.32/L) and an estimated minimum amount of drinking and cooking water needed for survival (15 L/person/day), the average sample household relying entirely on bottled water (2.72 persons, $50,195 income) was estimated to spend $4757 or 9.50% of their income on bottled water [34]. Despite these costs, Latinx Milwaukee parents have reported bottled water expenditure comprising up to 12.00% of their household income (median spending: 1.00% of household income) [31]. Moreover, 14.00% of Latinx parents reported having to sacrifice other purchases to afford bottled water [31]. Greater reliance on bottled water despite an added economic burden may be related to perceptions of unsafe tap water. 

Racial and ethnic differences in PWI choices are widely believed to be related to tap water safety perceptions and beliefs [10,11,12]. While perceptions were not evaluated via NHANES, there is considerable evidence suggesting the Latinx community has a greater mistrust of tap water quality and safety. Perceptions of tap water safety in US adults have been evaluated via cross-sectional analyses of the 2010 HealthStyles Survey (HSS, *n* = 3787) [35] and the nationally representative American Housing Survey (AHS) in 2013 (*n* = 126,424) [36] and 2015 (*n* = 39,085) [34]. Perceptions of parents of children and/or adolescents in various healthcare settings have also been evaluated cross-sectionally in smaller, regional investigations (Table 3). Prevalence of mistrust was similar across samples, representing 13.0% of the HSS [35], 9.2% of the 2013 AHS [36], and 7.3% of the 2015 AHS [34]. In both AHS samples, the prevalence of mistrust was greatest among Hispanic households (2013: 14.7%, 2015: 16.4%) and lowest among NH white households (2013: 5.2%, 2015: 5.1%) [34,36]. Hispanic households in 2015 were significantly less likely to trust the safety of their tap water compared with NH white households (OR = 0.406, S.E. = 0.0310, *p* < 0.01) [34]. However, prevalence was most pronounced in NH black adults (19.9%) in the HSS sample compared to 16.0% of Hispanic parents and 10.8% of NH white parents (*p* < 0.001) [35]. Perceptions were also assessed in a sample of US Hispanic adults (*n* = 1000) via the 2015 *Estilos* survey. Prevalence of mistrust was greater in this sample, in which 33.8 ± 2.6% of respondents did not believe their home tap water was safe to drink and 40.6 ± 2.8% did not believe their community tap water was safe to drink [37]. Among HSS Hispanic adults, the odds of low PWI (≤1 time/d) were significantly greater for those who did not trust tap water safety compared to those who did trust the safety or felt neutral about it (OR = 1.9, 95% CI = 1.1–3.5) [35]. The odds of low PWI were not different between perceptions of bottled water safety among Hispanic adults [35]. PWI in the month prior to the *Estilos* survey was not related to any of the drinking water perceptions in Hispanic adults [37]. 

Some studies have also assessed whether participants perceived bottled water to be safer than tap water. This belief was reported by 26.4% of HSS adults [35] and 26.0% of a sample of California adults (*n* = 306) via the 2011 Santa Clara County Dietary Practices Survey (SCCDPS) [38]. Prevalence was much higher among Hispanic respondents to the *Estilos* survey (64.7 ± 2.8%) [37]. Furthermore, 71.0% of the SCCDPS sample primarily consumed tap water (filtered or unfiltered) [38]. Accordingly, those who believed bottled water is safer were less likely to primarily consume tap water (OR = 0.28, 95% CI = 0.12–0.62, *p* = 0.002) [38]. Hispanic adults were significantly less likely to primarily consume tap water compared to NH white adults (OR = 0.33, 95% CI = 0.11–0.99, *p* = 0.48), only when the perception of safety was included in the model [38]. Additionally, adolescents and caretakers of children in academic community hospitals in Pennsylvania rated the safety of unfiltered tap water, filtered tap water, and bottled water as 3.0, 3.8, and 4.4 out of 5.0, respectively (*p* < 0.01), with a higher number indicating a more positive perception of quality [39]. Caretakers reported that infant formula was prepared exclusively with tap water (30.0%), exclusively with bottled water (51.0%), or with both (19.0%) [39]. Among caretakers using tap water for formula preparation, boiled tap water was most prevalent (54.0%) compared to unfiltered tap water (39.0%) and filtered water (7.0%) [39]. This suggests that most caretakers using tap water did not trust the safety of the tap water.

Findings from race/ethnicity comparisons are limited and inconsistent. The odds of thinking bottled water is safer than tap water were not significantly different between Hispanic and NH white adults (OR = 0.50, 95% CI = 0.11–2.27, *p* = 0.366) in the SCCDPS [38]. However, the prevalence of this perception in the HSS was greatest in NH black adults (40.00%) compared to 34.1% Hispanic parents and 21.8% of non-Hispanic white parents (*p* < 0.001) [35]. This was also found among parents of children in a US urban/suburban pediatric emergency department (*n* = 632), in which Latino parents were more likely to endorse a higher level of agreement with the statement “bottled water is safer than tap water” (20.00% vs. 9.3%, *p* < 0.001) [31]. In this sample, 44.8% of respondents gave their children primarily or exclusively bottled water [31]. Prevalence was significantly greater in Latino parents (~ 45%) than non-Latino white parents (~35%) (*p* < 0.001) [31]. Those who believed bottled water is safer than tap water were significantly more likely to primarily give their children bottled water (OR = 2.44, 95% CI = 1.44–4.22) [31]. Similarly, among parents of children in an urban public health center in Utah, 30.1% did not drink any tap water, and 41.20% reported never giving tap water to their child(ren) [40]. Of those children who did not drink tap, 59.6% exclusively consumed bottled water, while 35.6% exclusively consumed filtered water [40]. The odds of Latino parents consuming any tap water (OR = 0.26, 95% CI = 0.10–0.67) or giving their child(ren) tap water (OR = 0.32, 95% CI = 0.15–0.70) were significantly lower compared to non-Latino parents [40].

While there are limited and inconsistent findings regarding PWI and perceptions in Latinx adults, PWI source choices (tap vs. bottled) do appear to be related to perceptions of tap and bottled water safety. These behaviors may also translate into PWI sources for children and adolescents. However, PWI has only been measured through one unvalidated survey question about the frequency of consumption [35,37], through a question regarding the participant’s primary source of PWI [34,38,39,40], or not at all [31,36] (Table 3). Future work is needed that evaluates PWI and hydration status using validated methods, as well as perceptions, to provide more insight into these associations. Among these investigations that have quantitatively assessed tap water safety perceptions, predictors of perceptions have not consistently or comprehensively been evaluated (Table 4). We have compiled their findings to describe the complex, interrelated determinants of tap water safety perceptions in Latinx adults in the United States. Perceptions of unsafe tap water can occur in both the presence and absence of water insecurity. The predictors discussed in Section 3.2,Section 3.3,Section 3.4 and Section 3.5 are described in a context of water security. 

### 3.1. Water Insecurity

Current water insecurity is likely to impact tap water perceptions. Water security is largely out of US citizens’ hands, as much of the responsibility lies in the private and public provision and regulation of water (e.g., laws and regulations, water testing, water treatment, reporting, infrastructural maintenance, agricultural practices, etc.) [41,42]. Numerous factors, including historical planning processes, redlining, reduced enforcement of water regulations and standards, and repeat water violations contribute to a greater risk of water insecurity in minority and low-income communities [42]. 

Conflicting results have been observed regarding the association between geographic region and perceptions. This could be related to both rural and urban areas posing risks for water insecurity. Residents in rural areas are more dependent on wells, which have greater risks of water shortages and contamination [41]. On the other hand, 73.0% of households without piped water in the US (2013–2017) were located in urban areas. Moreover, individuals of color, with low-income, and with >30.0% of their income allocated to rent or mortgages or living in mobile homes, were more likely to not have access to piped water [43]. Prevalence of tap and bottled water trust were different between regions among the HSS survey sample [35] but not among the *Estilos* survey sample [37] (Table 5). The Hispanic sample in the *Estilos* survey had a lower prevalence of tap water safety trust and a greater prevalence of beliefs that bottled water is safer than tap water in all regions compared to the HSS sample. While differences in perceptions were not observed in the *Estilos* survey, agreement with the statement “I would buy less bottled water if I knew my local tap water was safe” was greatest among Hispanic adults in the South [37].

Influences of the built environment on tap water perceptions in the US have only been evaluated using the 2013 and 2015 AHS [34,36]. Greater overall housing unit quality, as rated by survey interviewers, was associated with increased odds of trusting tap water safety (2013: OR = 1.20, *p* ≤ 0.01; 2015: OR = 1.069, *p* < 0.01) [34,36]. However, inconsistent associations have been observed with more specific indicators of housing unit quality. The odds of trusting tap water safety were significantly lower among those living in a mobile home park (2013: OR = 0.64, *p* ≤ 0.01; 2015: OR = 0.738, *p* < 0.01) or living in public housing (2013: not included in model; 2015 OR = 0.764, *p* < 0.05) [34,36]. Both types of housing units are at greater risk for inadequate regulation of water systems. Furthermore, small, private water systems (i.e., serving < 15 households) are less regulated than public water systems and often source water from wells, which are prone to contamination [34,36]. Surprisingly, households with small water systems were more likely to trust their tap water (OR = 1.30, *p* ≤ 0.01) [36] in 2013, while perceptions did not differ between households with private wells or publicly regulated systems in 2015 [34]. Housing unit age (2013 AHS) also did not impact tap water safety perceptions [36]. Houses built after 1986 were proposed to have better water quality due to an amendment to the Safe Drinking Water Act that restricted the use of plumbing materials containing lead [36,44]. Finally, favorable perceptions of neighborhood quality were also associated with greater odds of trusting tap water safety (2013: OR = 1.14, *p* ≤ 0.01; 2015 OR = 1.149, *p* < 0.01) [34,36]. Satisfaction with neighborhood conditions as well as access to, and quality of, public services may translate into satisfaction with locally sourced tap water. Household income may also serve as an indicator of the household drinking water system and built environment, as income may determine the reliability of water utilities, quality of housing units, and quality of plumbing infrastructure [36]. 

Latinx individuals who do not trust their tap water safety may be more likely to seek alternatives. Modifications to the household water system can be implemented via home water treatment devices (i.e., carbon, fiber, reverse osmosis, neutralizers, chemical feed-pumps, disinfection and softeners, and pitcher water filters). Among US Hispanic adults who did not trust their tap water, only 1.9% drank unfiltered tap water, compared to 22.1% who consumed filtered tap water and 73.8% who consumed bottled water [34]. The risk of relying on unfiltered tap water (RR = 0.213, SE = 0.0798, *p* < 0.01) or filtered tap water (RR = 0.651, SE = 0.104, *p* < 0.01) over bottled water was significantly lower in Hispanic households compared to NH white households [34]. Among 2007–2010 NHANES cohorts, 33.1 ± 1.7% of adults utilized a home water treatment device [12]. Devices were utilized by 38.9 ± 2.4% NH white adults, 11.5 ± 1.4% of NH black adults, and 18.9 ± 1.3% Hispanic adults (*p* < 0.001) [12]. While perceptions were not measured, adults without water treatment devices were less likely to consume any plain water (OR = 0.60, 95% CI = 0.51, 0.71) or tap water (OR = 0.55, 95% CI = 0.47, 0.64) but more likely to consume bottled water (OR = 1.21, 95% CI = 1.01, 1.44) compared to adults with water treatment devices [12]. After adjustment for the use of a water treatment device, Hispanic adults were more likely to consume plain water (OR = 1.24, 95% CI = 1.03, 1.52) [12]. However, Hispanic adults were still less likely to consume tap water (OR = 0.61, 95% CI = 0.44, 0.83) and more likely to consume bottled water (OR = 1.89, 95% CI = 1.42, 2.52) compared to NH white adults [12]. Surveys of municipal water consumers in Florida revealed that water filters were only believed to adequately address organoleptics (i.e., bad-smelling water), whereas bottled water was preferred for more serious concerns (i.e., safety, contamination, and health risk) [45]. Therefore, while filtered water may be trusted more than unfiltered water, filtered water still may not be perceived as safe.

### 3.2. Individual Characteristics

Tap water trust appears to be greater with increased household income and education level but does not appear to be related to sex or age. Tap water safety was trusted by fewer Hispanic women (35.8 ± 3.7%) than men (43.4 ± 4.5%) who completed the *Estilos* survey (*p* = 0.08) [37]. Similarly, among participants who completed the 2015 AHS, women were significantly less likely to perceive tap water as safe compared to men (OR = 0.762, *p* < 0.01) [34]. However, sex differences were not observed in the HSS or SCCDPS [35,38]. No differences have been observed between age groups to date [35,37,38,39].

Based on the 2013 AHS, the odds of trusting tap water were not different for higher US household incomes per $1000 increase (OR = 1.00, *p* ≤ 0.01) [36]. However, the prevalence of trusting tap water significantly increased with increased income level in both the HSS and *Estilos* survey samples (Table 6) [35,37]. However, the prevalence of believing bottled water is safer than tap water significantly decreased with increased income level in the HSS sample [35] but was not different among the *Estilos* survey Hispanic sample [37]. This is particularly concerning for low-income individuals who spend a considerable proportion of their income on bottled water. Finally, among parents of children in an urban public health center in Utah (80.5% Latino), plain water preference (bottled vs. tap) was not associated with household income [40]. Even in the lowest income families (≤$14,999), 32.9% of parents exclusively gave their children bottled water while 32.0% exclusively gave their children filtered tap water [40].

Prevalence of trusting tap water among HSS adults was greatest in college graduates [35] (Table 7). This finding was supported by both AHS, in which US adults with at least a high school diploma were more likely to trust the safety of their tap water compared with those with less education (2013: OR = 1.448, S.E. = 0.108, *p* < 0.01 [34]; 2015: OR = 1.15, *p* ≤ 0.01 [36]). Among US Hispanic adults who completed the *Estilos* survey, tap water safety trust was more prevalent in adults with some college education and with a college degree [37]. An inverse association has been observed between education and perceptions of bottled water safety. Among the HSS sample, the prevalence of believing bottled water is safer than tap water was lowest in college graduates [35]. Similarly, California adults with at least some college education were less likely to think that bottled water was safer than tap water (OR = 0.32, 95% CI = 0.11–0.91, *p* = 0.033) compared with adults with a high school diploma or less [38]. However, among Hispanic adults who completed the *Estilos* survey, prevalence of beliefs that bottled water is safer than tap water was relatively high across all education levels [37]. Contrary to other samples, the greatest prevalence of this belief was observed in those with a college degree. 

### 3.3. Prior Experience with Poor Tap Water Quality

Latinx adults’ perceptions of tap water in the US may be influenced by prior experience with poor water quality in their home countries. US citizens are more likely to trust tap water than non-US citizens (OR = 1.556, SE = 0.134, *p* < 0.01) [34]. Similarly, 93.5% of native-born adults trust their tap water compared with 81.2% of foreign-born adults [36]. Among foreign-born adults, 70.9% of adults born in Latin American countries trusted tap water safety compared with 86.4% of adults born in all other world regions [36]. Among native-born race/ethnicity groups, Hispanic adults (of any race) had the lowest prevalence of trust (85.3%) [36]. Compared with non-Latino white households, there were significantly lower odds of perceiving tap water as safe in foreign-born (OR = 0.22, *p* ≤ 0.01) and native-born (OR = 0.41, *p* ≤ 0.01) Latino households [36]. For each additional year lived in a home country before residing in the US, the odds of trusting tap water were lower (OR = 0.99, *p* ≤ 0.01) [36]. However, US nativity did not influence the odds of primarily consuming tap water (filtered or unfiltered) or of believing bottled water is safer than tap water in California adults [38].

Experiences of immigrants may also influence second-generation Latinx adults’ perceptions [36]. Mistrust of home tap water safety was most prevalent among Latinx adults unacculturated to the US/English culture [37]. Among those assimilated to US/English culture, 18.2 ± 4.5% mistrust while 60.6 ± 6.7% trust tap water safety. Alternatively, prevalence of mistrust and trust are similar among bicultural adults (38.6 ± 3.8% vs. 33.5 ± 3.5%) and adults unaccultured to US/English culture (39.6 ± 5.0% vs. 31.0 ± 5.2%). Acculturation was scored based on years in the US, language spoken at home, cultural self-identification, and use of Spanish language media [37]. Among parents of children in an urban/suburban pediatric emergency department, the prevalence of prior bad experiences with bottled or tap water was similar across Latino, non-Latino whites, and African American parents [31]. However, the odds of primarily using bottled water (over tap) were significantly increased with having a bad experience with tap water (OR = 1.63, 95% CI = 1.06–2.46) and significantly decreased with primarily using tap water when younger (OR = 0.44, 95% CI = 0.23–0.83) [31]. Time spent living outside of the US at any time and level of education were not associated with the tendency to primarily use bottled water. 

### 3.4. Organoleptic (Sensory) Perceptions

Adolescents and caretakers have rated taste (4.5/5.0), clarity (4.8/5.0), and purity (4.2/5.0) highest in bottled water compared to filtered tap water (taste: 3.7/5.0, clarity: 4.6/5.0, purity: 3.5/5.0) and unfiltered tap water (taste: 3.0/5.0, clarity: 3.6/5.0, purity: 2.8/5.0) (*p* <0.01) [39]. Ratings of purity and safety were correlated for filtered tap water (r = 0.83) and bottled water (r = 0.78) [39]. Similarly, the odds of parents primarily relying on bottled water were significantly greater with beliefs that bottled water is cleaner (OR = 2.00, 95% CI = 1.14–3.51) and tastes better (OR = 2.76, 95% CI = 1.78–4.28) than tap water [31]. Beliefs about minerals and nutrients in tap and bottled water were not associated with primarily using bottled water. Furthermore, Latino parents were significantly more likely than non-Latino white parents to believe bottled water is cleaner (23.7% vs. 11.6%, *p* < 0.001), tastes better (24.8% vs. 14.2%, *p* < 0.001), and has more minerals and nutrients (9.0% vs. 0.9%, *p* < 0.001) than tap water [31]. Among another sample of parents, 30.6% of Latino parents and 25.6% of non-Latino parents avoided tap water because of taste [40].

It appears that taste, odor, and appearance of water may all be associated with health risk, even though organoleptics are not dependable indicators of health [46]. A previous study asked consumers to evaluate organoleptics of two blackcurrant juices and then to identify which juice they believed to be the most healthy [47]. Overall impressions (general sensory appeal) of each juice were highly correlated with flavor ratings and the frequency with which they would consume each juice [47]. Moreover, a majority of consumers believed that the juice they rated highest for “overall impression” was also the healthiest juice [47]. Thus, consumers may also associate organoleptics of water, particularly taste, with health and subsequently with safety. Furthermore, tap water consumers have been found to rate organoleptics (i.e., taste, odor, and color) as well as health and quality/hygiene of tap water to be significantly more preferable to that of bottled water [48]. On the other hand, bottled water consumers rated bottled water as significantly more preferable than tap water for the same factors [48]. Interestingly, blind taste-tests in the second sample of German students revealed that taste and health preferences diminished when they were blinded to water sources [48]. 

Conversely, negative organoleptic perceptions may be interpreted as a health risk. Latino parents of young children residing in a rural California community participated in focus groups and qualitative interviews [49]. They reported believing their tap water was not safe to drink because of taste (i.e., salty, strongly of chlorine), appearance (i.e., brown, yellow), and a smell that they associated with adverse health concerns (i.e., stomach aches, nausea, vomiting, skin irritations/lesions, hair loss) [49]. They also believed that their children were at a greater risk since they were still developing [49]. Similarly, participants living in an under-resourced rural area in New Mexico reported during focus groups that their community’s water was unappealing, dirty, and unsafe [30]. They also believed that chlorine or other minerals contributed to the bad taste of their tap water and were opposed to drinking it or bathing or cooking with it [30]. Perceptions of water quality (i.e., bad taste, discoloration) contributed to perceptions that tap water was not safe [30].

### 3.5. Availability and Sources of Information

Few studies have assessed where individuals receive information about tap water. Among parents of children in an urban/suburban pediatric emergency department, the news, advertising, friends, and physicians were similarly prevalent sources of information about water across race/ethnicity groups [31]. However, family as a source of information was more prevalent among Latino parents than non-Latino white parents (*p* < 0.01) [31]. Additionally, environmental organizations were less common sources of information for Latino parents, though not significantly so (*p* = 0.06). Receiving information on tap water from an environmental organization was associated with greater odds of primarily relying on bottled water (over tap water) (OR = 1.74, 95% CI = 1.03–2.93) [31]. Finally, avoiding tap water because someone told them not to drink it was not prevalent among Latino (8.1%) or non-Latino (4.7%) parents of children in an urban public health center in Utah [40].

The United States Environmental Protection Agency (EPA) requires public water systems to inform citizens about the quality of their local water annually via consumer confidence reports (CCRs) [50]. However, these reports may not meet the readability standard for most of the national population (i.e., written at the 6th–7th-grade level) [51,52]. A nationally representative set of CCRs from 2011–2013 was determined to be written at the 11th–14th-grade level [51]. The style was considered difficult, similar to that of academic and scientific publications (e.g., *Harvard Law* Review articles) and requiring high school or some college education for comprehension [51]. Consequently, education level may impact perceptions through accessibility, understanding, and confidence in information on water safety and quality [34]. 

Bottled water advertisements and marketing may also contribute to water-related perceptions and behaviors [36,53]. Bottled water marketing campaigns have commonly utilized labels such as pure, pristine, natural [54], and healthy to promote positive associations with bottled water health, risk, and organoleptic perceptions [53]. The influence of marketing tactics has been observed as college students’ intention to purchase bottled water was related to its perceived benefits (e.g., convenience, taste, and health) [55]. Bottled water marketing and advertising campaigns also have a history of targeting minorities [34]. Furthermore, the odds of bottled water reliance in parents of children treated in an urban/suburban pediatric emergency department were significantly greater for those who believed their “family may be protected from illness by choosing the best kind of drinking water” (OR = 1.53, 95% CI = 1.01–2.32) [31]. Latino and African American parents were more likely to strongly agree with that statement compared to NH white parents (*p* = 0.007) [31]. Similarly, 42.2% of Latino parents of children cared for in an urban public health center reported avoiding tap water because it caused illness [40]. Latino parents were significantly more likely than non-Latino parents to avoid tap water due to fear of illness (OR = 5.63, 95% CI = 2.17–14.54) [40]. This is in contrast to testimony from the US Government Accountability Office concluding that while regulations for tap water by the EPA and bottled water by the US Food and Drug Administration are similar, the US Food and Drug Administration lacks the authority to enforce them [39,56]. Specifically, bottled water is not required to be tested in certified laboratories, and findings from testing, including violations of water quality standards, are not required to be reported [56]. Furthermore, bottled water labels are not required to include any information on compliance with regulations or contaminants present in their water and their potential health risks [56]. Positive perceptions of bottled water via marketing and advertising combined with a lack of information about bottled water safety may reinforce negative perceptions about tap water. 

Latinx distrust in CCRs and trust in the bottled water industry could also be related to overall distrust in the government. Latino parents of young children residing in a rural California community, who were primarily low-income and of low education level, reported not trusting their tap water safety due to a history of municipal water quality problems in the community [49]. These beliefs were even held by newer residents, who were warned by long-term residents, despite recent infrastructural improvements implemented by the government in addition to regular testing conducted by an institution independent of the government [49]. While they reported not being aware of CCRs, they did believe independent water testing would convince them of tap water safety [49]. Although there have been inconsistent findings regarding Latino adults’ trust in the US government [57,58], distrust in Mexican Americans has been observed to increase with acculturation into American culture as well as with experience with and/or observation of racism and discrimination [58].

## 4. Conclusions

Adherence to IOM adequate intake recommendations for TWI has been low in recent years. TWI has been consistently lower in Latinx adults compared to NH white adults. The decision to drink water is complex and is influenced by a myriad of factors including context, environment, eating behaviors, geography, and beverage attributes. While overall PWI is similar between Latinx and NH white adults, Latinx adults are particularly averse to tap water. Thus, voluntary low TWI in Latinx adults appears to be driven by tap water avoidance. Tap water perceptions are complex and appear to be influenced by water insecurity, demographics, prior experiences, organoleptic (sensory) perceptions and availability and sources of information. Existing interventions designed to improve TWI primarily focus on improving access to water and/or educating individuals on the importance of hydration. However, this may not be sufficient in Latinx populations where water is not trusted. Furthermore, while overall PWI is similar across races and ethnicities, overall TWI is low and it’s important for individuals to increase TWI through PWI and not through other beverages. Trust in tap water (in water secure contexts) could improve access to and convenience of water and improve the likelihood of choosing water over other beverages. Future work should comprehensively assess these factors in Latinx samples and include validated PWI, TWI, and hydration status measures. A greater understanding of these relationships could inform interventions to improve TWI and hydration status in Latinx adults. 

## Figures and Tables

**Table 1:** Total water intake and percentage of individuals meeting IOM adequate intake recommendations for water by age group from recent NHANES cohorts.

NHANES Years	Measures	Age Groups			
2005–2010 [10]		20–50 y (*n* = 8389)	51–70 y (*n* = 4737)	≥71 y (*n* = 2576)	
	Total water intake (mL) ^1,2^	3560 ± 30	3229 ± 27	2251 ± 17	
	Men meeting IOM recommendations (%)	57.3	40.9	5.3	
	Women meeting IOM recommendations (%)	59.4	55.1	17.4	
2011–2016 [13]		19–30 y (*n* = 3248)	31–50 y (*n* = 5071)	51–70 y (*n* = 4873)	>70 y (*n* = 2071)
	Total water intake (mL) ^1,2^	2936 ± 52	3166 ± 36	2997 ± 43	2355 ± 28
	Men meeting IOM recommendations (%) ^3^	~36.0	~41.0	~32.0	~5.0
	Women meeting IOM recommendations (%) ^3^	~44.0	~56.0	~53.0	~24.0

Abbreviations: IOM, Institute of Medicine; NHANES, National Health and Nutrition Examination Survey. ^1^ Data are presented as mean ± standard error; ^2^ Total water intake refers to the total amount of water consumed via plain water, beverages, and water of solid food; ^3^ Values were determined from visual inspection of a figure

**Table 2:** Total water intake and plain water intake by race/Hispanic origin from recent NHANES cohorts ^1^.

NHANES Years	Race/Hispanic Origin	Tap Water Intake (mL)	Bottled Water Intake (mL)	Plain Water Intake (mL) ^2^	Total Water Intake (mL) ^3^
2005–2010 [10]	Non-Hispanic White (*n* = 7610)	703 ± 17	437 ± 12	1134 ± 19	3439 ± 24
	Mexican American (*n* = 2899)	383 ± 22 *	729 ± 33 *	1095 ± 25	3037 ± 36 *
	‘Other’ Hispanic (*n* = 1322)	455 ± 35 *	758 ± 48 *	1208 ± 41	3156 ± 44 *
2009–2012 [11]	Non-Hispanic White (*n* = 3541)	828 ± 47 ^4^	379 ± 24 ^4^	1183 ± 47 ^4^	3341 ± 53
	Hispanic (*n* = 2048)	544 ± 47 ^4^*	710 ± 47 ^4^*	1207 ± 47 ^4^	3005 ± 57 *
2011–2014 [12]	Non-Hispanic White (*n* = 5277)	813 ± 38	345 ± 19	1158 ± 34	NR
	Hispanic (*n* = 3095)	550 ± 40 *	731 ± 39 *	1281 ± 48 *	NR

Abbreviations: NHANES, National Health and Nutrition Examination Survey; NR, not reported. ^1^ Data are presented as mean ± standard error; ^2^ Plain water intake refers to the total amount of water consumed via tap water and bottled water; ^3^ Total water intake refers to the total amount of water consumed via plain water, beverages, and water of solid food; ^4^ Values were converted from # 8-fl oz servings to mL (29.57 mL·fl oz^−1^). * Significantly different from non-Hispanic white adults (*p* < 0.05).

**Table 3:** Investigational approaches to measuring plain water intake and perceptions.

Author, Year	*n*	Sample	Plain Water Intake Measurement	Perception Measurement
Park et al., 2019 [37]	1000	US Hispanic adults (≥18 y)	*Estilos* Survey Fall 2015: 1. During the past month, how often did you drink a glass or bottle of plain water? Include tap, water fountain, bottled, and unflavored sparkling water Response options: none, 1–6 times/wk, 1 time/d, 2 times/d, 3 times/d, ≥4 times/d	*Estilos* Survey Fall 2015: 1. My tap water is safe to drink 2. Community tap water is safe to drink 3. Bottled water is safer than tap water 4. I would buy less bottled water if my tap water was safe Response options: strongly disagree, somewhat disagree, neither agree nor disagree, somewhat agree, strongly agree
Javidi et al., 2018 [34]	39,085	Sample of households nationally representative of US housing stock	2015 American Housing Survey: 1. Where do you get your water for drinking? *asked only if answered no to “In your opinion, is the water from this source for cooking and drinking?” Response options: unfiltered tap water, filtered tap water, commercial bottled water, other	2015 American Housing Survey: 1. In your opinion, is the water from this source [housing unit] safe for cooking and drinking? Response options: Self-reported response recoded as binary variable – yes or no
Pierce et al., 2017 [36]	126,424	Sample of households nationally representative of US housing stock	-	2013 American Housing Survey: 1. In your opinion, is the water from this source [housing unit] safe for cooking and drinking? Response option: Self-reported response recoded as binary variable – yes or no
van Erp et al., 2014 [38]	306	Adults (≥18 y) in Santa Clara County, California	2011 Santa Clara County Dietary Practices Survey: 1. Report the type of water consumed most often on a typical day Response options: format is not clear – responses categorized as primarily drinks tap water (unfiltered tap or filtered tap) or primarily drinks bottled plain water or seltzer (soda) water	2011 Santa Clara County Dietary Practices Survey: 1. Which do you think is safer, bottled water or Santa Clara County tap water or are they about the same? Response options: format is not clear – responses categorized as thinks bottled water is safer or does not think bottled water is safer
Onufrak et al., 2012 [35]	3787	US respondents to ConsumerStyles survey (consumer mail survey) (≥ 18 y)	2010 HealthStyles Survey: 1. On a typical day, how many times do you drink a glass or bottle of plain water? count tap, bottled, and unflavored sparkling water. Response options: none, 1 time/d, 2 times/d, 3 times/d, 4 times/d, ≥ 5 times/d *Low intake = ≤ 1 time/d*	2010 HealthStyles Survey: 1. My local tap water is safe to drink 2. Bottled water is safer than tap water Response options: strongly disagree, somewhat disagree, neither agree nor disagree, somewhat agree, strongly agree
Huerta-Saenz et al., 2012 [39]	208	Caretakers of children and adolescents in an academic community hospital in Pennsylvania	14-Question Survey ^1,2,4^: 1. Preferred type of drinking water 2. Preferred type of water used for cooking Response options: filtered tap water, unfiltered tap water, bottled water, do not drink water	14-Question Survey ^1,2,4^: 1. Taste of tap (filtered and unfiltered) and bottled water 2. Safety of tap (filtered and unfiltered) and bottled water 3. Clarity of tap (filtered and unfiltered) and bottled water 4. Purity of tap (filtered and unfiltered) and bottled water Response options: Rate items on a 5-pt Likert scale [5 highest]
Gorelick et al., 2011 [31]	632	Parents of children treated at an urban/suburban emergency department in Milwaukee, Wisconsin	-	Questionnaire ^1,3,4^: (*11 belief statements, 4 statements about prior water use experiences, 7 statements about sources of information on tap and bottled water*) 1. Bottled water is cleaner than tap water 2. Bottled water is safer than tap water 3. Bottled water tastes better than tap water 4. Bottled water is more convenient than tap water 5. Bottled water has minerals and nutrients that tap water does not 6. My family may be protected from illness by choosing the best kind of drinking water Response options: Agreement for each statement rated on 5-point Likert scale [1, strongly agree; 5, strongly disagree]
Hobson et al., 2007 [40]	216	Parents of children attending an urban public health clinic in Utah	15-Question Survey ^1,4^: 1. Do you drink tap water at home? 2. Do you give tap water at home to your children? 3. If your children drink tap at home, is it filtered? 4. Do your children drink bottled water at home? Response options: always, sometimes, never 5. What type of filter do you use? Response options: Water pitcher, faucet mounted, under sink [reverse osmosis or distillation], I don’t know	15-Question Survey ^1,4^: 1. If your child does not drink tap water at home, why not? Response options: I don’t know how it tastes, I think tap water will make me sick, I was told not to drink tap, other

^1^ Survey/questionnaire created by investigators; ^2^ Survey/questionnaire pilot-tested by investigators; ^3^ Survey/questionnaire created based on semi-structured interview; ^4^ Only some questions/statements from survey/questionnaire included in publication.

**Table 4:** Predictors included in investigations evaluating perceptions of tap water safety in US Latinx adults.

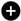 Included in Investigation	Park et al. (2019) [37]	Javidi et al. (2018) [34]	Pierce et al. (2017) [36]	van Erp et al. (2014) [38]	Onufrak et al. (2012) [35]	Huerta-Saenz et al. (2012) [39]	Gorelick et al. (2011) [31]	Hobson et al. (2007) [40]
Geographic region	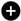				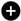			
Household and neighborhood characteristics		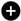	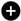					
Household income	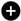		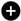	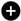	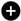		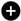	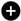
Education level	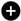	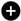	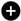	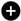	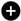		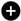	
Sex	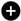	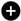		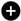	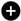			
Age	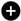			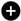	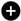	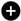		
Race/Ethnicity		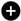	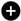	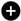	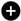	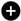	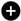	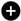
US nativity		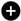	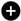	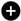			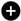	
Had a bad experience with tap water							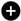	
Organoleptic (sensory) factors						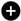	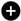	
Sources of information about water							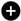	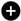

**Table 5:** Prevalence of agreement with perceptions by geographic region among survey samples.

Survey	Perception	Geographic Region								
HSS [35]		New England	Middle Atlantic	South Atlantic	East South Central	West South Central	East North Central	West North Central	Mountain	Pacific
	“My local tap water is safe to drink” *	77.4%	64.2%	66.8%	68.7%	59.0%	70.9%	77.3%	71.0%	65.6%
	“Bottled water is safer than tap water” *	35.1%	22.9%	27.1%	25.0%	34.6%	27.2%	13.4%	24.1%	30.5%
*Estilos* [37]		Northeast		South			Midwest		West	
	“My tap water at home is safe to drink” ^1^	35.7 ± 5.9%		42.3 ± 5.6%			29.7 ± 7.8%		40.9 ± 4.4%	
	“Bottled water is safer than tap water”^1^	62.9 ± 6.4%		61.2 ± 5.5%			61.8 ± 8.5%		69.2 ± 4.0%	
	“I would buy less bottled water if I knew my local tap water was safe” ^1^*	63.0 ± 6.0%		74.9 ± 4.70%			59.7 ± 8.7%		67.2 ± 4.1%	

Abbreviations: HSS, HealthStyles Survey. ^1^ Data are presented as mean ± standard error. * Significantly different prevalence by geographic region (*p* < 0.05).

**Table 6:** Prevalence of agreement with perceptions by household income level among survey samples.

Survey	Perception	Income Level			
HSS [35]		<$25,000	$25,000–$59,999	≥$60,000	
	“My local tap water is safe to drink” *	59.4%	68.8%	71.8%	
	“Bottled water is safer than tap water” *	34.3%	22.9%	24.8%	
*Estilos* [37]		≤$24,999	$25,000–$44,999	$45,000–$69,000	≥ $70,000
	“My tap water at home is safe to drink” ^1^*	24.4 ± 3.0%	35.7 ± 5.6%	41.1 ± 7.2%	58.1 ± 6.3%
	“Bottled water is safer than tap water” ^1^	63.6 ± 4.0%	65.5 ± 5.4%	60.1 ± 7.2%	68.2 ± 6.5%

Abbreviations: HSS, HealthStyles Survey. ^1^ Data are presented as mean ± standard error. * Significantly different prevalence by income level (*p* < 0.05).

**Table 7:** Prevalence of agreement with perceptions by education level among survey samples.

Survey	Perception	Education Level			
HSS [35]		<High School Degree	High School Degree	Some College Education	College Degree
	“My local tap water is safe to drink” *	63.3%	65.5%	62.1%	77.5%
	“Bottled water is safer than tap water” *	40.0%	27.5%	29.5%	19.9%
*Estilos* [37]	“My tap water at home is safe to drink” ^1^*	27.3 ± 5.0%	37.2 ± 6.1%	53.3 ± 5.5%	52.1 ± 5.9%
	“Bottled water is safer than tap water” ^1^*	61.8 ± 5.3%	64.5 ± 6.1%	55.1 ± 5.7%	78.2 ± 3.4%

Abbreviations: HSS, HealthStyles Survey. ^1^ Data are presented as mean ± standard error. * Significantly different prevalence by education level (*p* < 0.05).

## Data Availability

No new data were created or analyzed in this study. Data sharing is not applicable to this article.
